# ERα is required for suppressing OCT4‐induced proliferation of breast cancer cells via DNMT1/ISL1/ERK axis

**DOI:** 10.1111/cpr.12612

**Published:** 2019-04-22

**Authors:** Xiangshu Jin, Yanru Li, Yantong Guo, Yiyang Jia, Huinan Qu, Yan Lu, Peiye Song, Xiaoli Zhang, Yijia Shao, Da Qi, Wenhong Xu, Chengshi Quan

**Affiliations:** ^1^ The Key Laboratory of Pathology, Ministry of Education, College of Basic Medical Science Jilin University Changchun China

**Keywords:** breast cancer, DNMT1, ERK, ERα, ISL1, OCT4, proliferation

## Abstract

**Objective:**

POU5F1 (OCT4) is implicated in cancer stem cell self‐renewal. Currently, some studies have shown that OCT4 has a dual function in suppressing or promoting cancer progression. However, the precise molecular mechanism of OCT4 in breast cancer progression remains unclear.

**Materials and Methods:**

RT‐PCR and Western blot were utilized to investigate OCT4 expression in breast cancer tissues and cells. Cell proliferation assays and mouse models were applied to determine the effects of OCT4 on breast cancer cell proliferation. DNMT1 inhibitors, ChIP, CoIP, IHC and ERα inhibitors were used to explore the molecular mechanism of OCT4 in breast cancer.

**Results:**

OCT4 was down‐regulated in breast cancer tissues, and the overexpression of OCT4 promoted MDA‐MB‐231 cell proliferation and inhibited the proliferation of MCF‐7 cells in vitro and in vivo, respectively. Two DNMT1 inhibitors (5‐aza‐dC and zebularine) suppressed OCT4‐induced MDA‐MB‐231 cell proliferation through Ras/Raf1/ERK inactivation by targeting ISL1, which is the downstream of DNMT1. In contrast, OCT4 interacted with ERα, decreased DNMT1 expression and inactivated the Ras/Raf1/ERK signalling pathway in MCF‐7 cells. Moreover, ERα inhibitor (AZD9496) reversed the suppression of OCT4‐induced proliferation in MCF‐7 cells via the activation of ERK signalling pathway.

**Conclusions:**

OCT4 is dependent on ERα to suppress the proliferation of breast cancer cells through DNMT1/ISL1/ERK axis.

## 1 INTRODUCTION

*OCT4* gene (official symbol *POU5F1*) is a mammalian POU transcription factor that affects the maintenance of self‐renewal and pluripotency, which are the central features of embryonic stem cells. Human *OCT4* gene can generate at least three transcripts (*OCT4A*, *OCT4B* and *OCT4B1*) and four protein isoforms (OCT4A, OCT4B‐190, OCT4B‐265 and OCT4B‐164) by alternative splicing and translation initiation. OCT4A (often referred to as OCT4) is a transcription factor that regulates stemness. While OCT4B cannot sustain ES cell self‐renewal, it may respond to cell stress. However, the function of OCT4B1 is still unclear.^1^ There are seven pseudogenes of human *OCT4* gene: *OCT4‐pg1*, *OCT4‐pg2*, *OCT4‐pg3*, *OCT4‐pg4*, *OCT4‐pg5*, *OCT4‐pg6* and *OCT4‐pg7*.^2^ Studies have revealed that transcription factors related to the maintenance of “stemness,” such as Sox‐2 and c‐Myc, which are involved in the suppression of cancer growth and metastasis,^3^, ^4^ and OCT4 could suppress the metastatic potential of breast cancer cells (BCCs).^5^ OCT4 has been used as a marker of cancer stem cells and has distinct functions in different pathways in cancer cells. Some studies have revealed that aberrant expression of OCT4 is linked to a variety of human cancers.^6^, ^7^, ^8^ Furthermore, epigenetic mechanisms, such as DNA modification, is involved in tumour‐propagating phenotype induced by OCT4.^9^ However, the role and underlying mechanism of OCT4 in BC progression remain elusive.

Human cancers have been found to be associated with aberrant DNA hypermethylation at CpG islands of tumour suppressor genes, most of which are unmethylated in normal cells.^10^ DNMT1 is a major DNA methyltransferase that is responsible for maintaining the methylation status during DNA replication.^11^ DNMT3a and DNMT3b mainly perform de novo methylation of either unmethylated DNA or hemimethylated DNA to assist in maintenance.^12^ Previous studies demonstrated that the stem cell pluripotent markers OCT4 and Nanog could regulate DNA methylation during differentiation in embryonic stem cells, and changes in DNA methylation patterns result in altered cell proliferation.^13^ Additionally, DNA methylation is associated with tumour proliferation and metastasis through the extracellular signal–regulated kinase (ERK) signalling pathway.^14^, ^15^ ERK1/2, also known as p42/44 mitogen‐activated protein kinase (MAPK), can be activated by a variety of growth factors and has many substrates. ERK signalling promotes the activated GTP‐bound Ras proteins to activate the Raf‐MEK‐ERK kinase cascade by a series of phosphorylation events of the kinases.^16^ The Ras‐ERK pathway mediates various cellular processes, including cell growth, proliferation, differentiation, survival and migration.^17^, ^18^ Our recent study using the next‐generation sequencing (NGS) showed that overexpression of OCT4 in human hair follicle mesenchymal stem cells up‐regulated the expression of 1181 genes, including *KRAS* gene, which is the upstream of ERK signalling pathway.^19^ Therefore, the correlation of the stem cell pluripotent marker OCT4, DNA methylation and ERK signalling pathway in breast cancer proliferation should be examined. However, the present studies demonstrate that OCT4 exerts dual effects in breast cancer,^5^, ^20^ which may be related to the multiple intrinsic genes involved in different breast cancer subtypes, especially estrogen receptor (ER), progesterone receptor (PR) and human epidermal growth factor 2 (HER2). Estrogen receptor alpha–positive (ERα+) subtype accounts for approximately 80% of all breast cancers, which is the most common cancer in women.^21^ Up to 50% of ERα+ primary BC lose ERα expression in recurrent tumours, conferring resistance to tamoxifen therapy.^22^ Inactivation of *ESR1* gene via methylation strongly correlates with poor prognosis as well as an aggressive phenotype in TNBC.^22^ Additionally, ERα can be complexed with OCT4 to promote tamoxifen resistance in breast cancer cells.^23^

In the current study, we provide evidence that OCT4 is down‐regulated in invasive breast cancer, which plays a key role in BCC proliferation. However, OCT4 can function as an oncogene as well as tumour suppressor gene in TNBCs and luminal A subtype cells. Therefore, we elucidated the mechanism by which OCT4 exerts its tumour‐suppressive function, showing that OCT4 is dependent on ERα to suppress the proliferation of breast cancer cells through DNMT1/ISL1/ERK axis, and this axis will be a novel potential target for improving the diagnosis, therapy and prognosis of breast cancer patients.

## 2 MATERIALS AND METHODS

### 2.1 Patient samples and cell culture

Paraffin‐embedded tissues, including normal breast tissues and breast cancer tissues, were collected from patients at the Second Hospital of Jilin University. The study was approved by the Ethics Committee of Jilin University (Changchun, Jilin, PR China). None of the patients received neo‐adjuvant therapy. The patients’ medical records were reviewed to obtain their age, tumour status and clinical stage. All cancer cases were classified and graded according to the International Union Against Cancer (UICC) staging system for breast cancer.

The human breast cancer cell lines MDA‐MB‐231 (triple‐negative type), MCF‐7 (luminal type) and SKBR3 (HER2 type) were cultured in Dulbecco's modified Eagle's medium (DMEM) (Gibco) supplemented with 10% foetal bovine serum (FBS; BI, Israel) at 37°C in a humidified 5% CO_2_ atmosphere.

### 2.2 Western blot analysis

Western blot analysis was conducted according to our previous protocol.^24^ The following antibodies were used: OCT4 (1:1000; Abcam, ab19857), β‐actin (1:2000; CST, #3700), DNMT1 (1:1000; Abcam, ab13537), Ras (1:1000; Abcam, ab52939), Raf1 (1:1000; Abcam, ab137435), P‐ERK (1:1000; CST, #4377s), ERK (1:1000; CST, #4695s), ER alpha (1:1000; CST, #8644s) and ISL‐1 (1:100; Abcam, ab178400).

### 2.3 Reverse transcription PCR

Total RNA was collected using TRIzol reagent (Invitrogen). Reverse transcription PCR (RT‐PCR) was conducted according to our previous protocol.^24^ GAPDH was used as an endogenous control. The PCR primers are shown in Table 1 and Table S1. The reaction products were resolved on 1.5% agarose gels and visualized by staining with ethidium bromide. The image was observed and photographed under a viltalight lamp using a Gel Imaging System (Bio‐Rad Laboratories, Inc, Hercules, CA). The results were analysed by Quantity One 4.4.1 software (Bio‐Rad Laboratories, Inc).

**Table 1:** PCR primers sequences

Gene name	Sequence 5′→3′
RT‐PCR primers	
*OCT4*	
Sense	CTGAAGCAGAAGAGGATCAC
Antisense	GACCACATCCTTCTCGAGCC
*ISL1*	
Sense	GCGGAGTGTAATCAGTATTTGGA
Antisense	GCATTTGATCCCGTACAACCT
*ESR1*	
Sense	TGATGAAAGGTGGGATACGAAA
Antisense	GGCTGTTCTTCTTAGAGCGTTTG
*GAPDH*	
Sense	CCATGTTCGTCATGGGTGTGA
Antisense	CATGGACTGTGGTCATGAGT
ChIP primers	
*DNMT1* (OCT4)	
Sense	CCCCACACACTGGGTATAGAA
Antisense	CGAGGCATTCATTCATTCATT
*DNMT1* (ERα)	
Sense	CAAGCCATCCTCCCACCTCAG
Antisense	CCAGCCTGAGCAACATAGGGATAC

### 2.4 Lentivirus production and lentivirus transduction

The lentivirus vector pLV‐EF1α‐OCT4‐IRES‐EGFP and packaging plasmids expressing gag‐pol, pVSVG and rev genes were obtained from the Institute of Biochemistry and Cell Biology of the Shanghai Life Science Research Institute, Chinese Academy of Science. These vectors were transfected into 293T cells by FuGene HD (Roche). Viral supernatants were harvested at 48 and 72 hour after transfection and concentrated by ultracentrifugation. MDA‐MB‐231 cells and MCF‐7 cells were seeded on 6‐well plates and infected with lentivirus expressing OCT4 in the presence of 5 mg/mL polybrene for 24 hours. Then, OCT4 expression in the cells was validated by PCR and Western blot.

### 2.5 Cell proliferation assay

Cell proliferation was assessed by counting cell numbers in real time using the iCELLigence Real‐Time Cell Analysis system (ACEA Biosciences) and E‐plates (Roche), which monitor cellular events in real time by measuring electrical impedance across interdigitated gold micro‐electrodes integrated on the bottom of cell culture plates. The impedance measurement provides quantitative information about the biological status of the cells, including cell number, viability and morphology.

### 2.6 Plate colony formation assay

In 6‐well plate, cells were seeded into each well with 2 mL DMEM supplemented with 10% FBS. After 2 weeks, the resulting colonies were fixed with methanol at room temperature for 15 minute and then stained with Giemsa for 20 minute. Colonies were counted. The colony formation index was defined as the ratio of colony numbers to the initial numbers of the cells plated in each well (100 cells/well).

### 2.7 Soft agar colony formation assay

Cell suspensions were mixed with 1.2% soft agar in DMEM containing 20% FBS. In 6‐well plate, cells were seeded into each well with 2 mL DMEM supplemented with 10% FBS. Next day, the cell suspension was removed. After 2 weeks, the colonies were counted. The colony formation index was defined as the ratio of colony numbers to the initial numbers of the cells plated in each well (100 cells/well).

### 2.8 Immunofluorescence

Cells were seeded on small coverslips. After washing three times with PBS, the cells were fixed with 4% paraformaldehyde for 10 minute at room temperature. The cells were incubated with 0.1% Triton X‐100 and BSA for 1 hour and then incubated with Ki67 antibody (1:200; Abcam, ab15580) at 4°C overnight. After washing three times with PBS, the cells were incubated with secondary antibody (1:1000; CST, #8889) for 1 hour. After washing three times with PBS, cells were stained with DAPI for nuclei (D8417; Sigma).

### 2.9 Animal model

Mice were purchased from Beijing Vital River Laboratory Animal Technology Company. Mice were housed under hygienic conditions according to the Chinese guidelines governing animal experimentation, and their care was in accordance with institutional guidelines. Animal experiments were approved by the Chinese veterinary authorities. All mice used in the study were 4‐ to 5‐week‐old female BALB/c nude mice. Each group had 5 mice. For subcutaneous injections, 5 × 10^6^ MDA‐MB‐231 cells (MDA‐MB‐231‐pEF as a control group and MDA‐MB‐231‐OCT4 cells) and 5 × 10^6^ MCF‐7 cells (MCF‐7‐pEF as a control group and MCF‐7‐OCT4 cells) in 100 μL PBS were injected into nude mice. After 3‐4 weeks, the primary tumour reached 1000 mm^3^ in volume or signs of distress were observed. Tumour volumes were determined according to the following formula: (length × width^2^)/2. After the last measurement of tumour volume, the mice were sacrificed under anaesthesia with 5 mg/100 g body weight sodium pentobarbital, and tumour tissues were removed.

### 2.10 Reagents

Cells were treated with various concentrations and durations of the DNA methyltransferase inhibitor zebularine (Sigma, Z4775) and 5‐aza‐dC (Sigma, A3656) and the estrogen receptor (ERα) antagonist AZD9496 (MCE, HY‐12870).

### 2.11 Co‐Immunoprecipitation

Cells were lysed in IP Lysis/Wash Buffer supplemented with phenylmethane sulfonyl fluoride (PMSF) and protease inhibitor cocktail (PIC) at 4°C for 30 minute and then centrifuged at 13 000 *g* for 20 minute at 4°C. Lysate was immunoprecipitated with the appropriate antibodies and Protein A/G Magnetic beads (Millipore) at 4°C for 6 hours. The beads were washed five times with IP Lysis/Wash Buffer. The immunocomplexes were eluted for 5 minute and analysed by Western blot. The antibodies used in this study were OCT4 (Abcam, ab19857) and ERα (Abcam, ab108398).

### 2.12 Immunohistochemistry

All samples were fixed in 4% paraformaldehyde overnight at 4°C and then dehydrated in different concentrations of ethanol. Immunohistochemistry staining and analytical methods were performed according to the protocol of the UltraSensitiveTM SP（Mouse/Rabbit）immunohistochemistry (IHC) kit (Maxim). Anti‐OCT4 (1:1000; Abcam, ab181557), ISL‐1 (1:200; Abcam, ab178400), ERα (1:200; Abcam, ab108398), PR (1:200; Abcam, ab2765), HER2 (1:200; Abcam, ab16662) and Ki67 (1:200; Abcam, ab15580) antibodies were used in the experiments.

### 2.13 Chromatin immunoprecipitation

Chromatin immunoprecipitation (ChIP) was performed using the EZ Magna ChIP G chromatin immunoprecipitation kit (Millipore). The genomic DNA of lysed cells was sheared to 200‐500 bp by sonication. The final lysate was incubated with OCT4‐, and ERα‐specific antibodies and precipitated with protein A/G magnetic beads. After three washes, DNA‐protein complexes were reversely cross‐linked, and genomic DNA was extracted with a Wizard Genomic DNA Purification kit (Promega), and eluted in 50 μL of TE buffer. After elution, quantitative PCR (qPCR) was performed to amplify the DNA fragment containing the OCT4 and ERα binding sites on the DNMT1 promoter. The qPCR primers are summarized in Table 1.

### 2.14 Statistical analysis

Differences between means of independent groups were tested using unpaired Student's *t* ‐test. Analyses were carried out using GraphPad Prism 7 (GraphPad software). *P* values < 0.05 were considered statistically significant. Each experiment was repeated three times.

## 3 RESULTS

### 3.1 OCT4 expression is down‐regulated in breast cancer tissues

**Figure 1:**
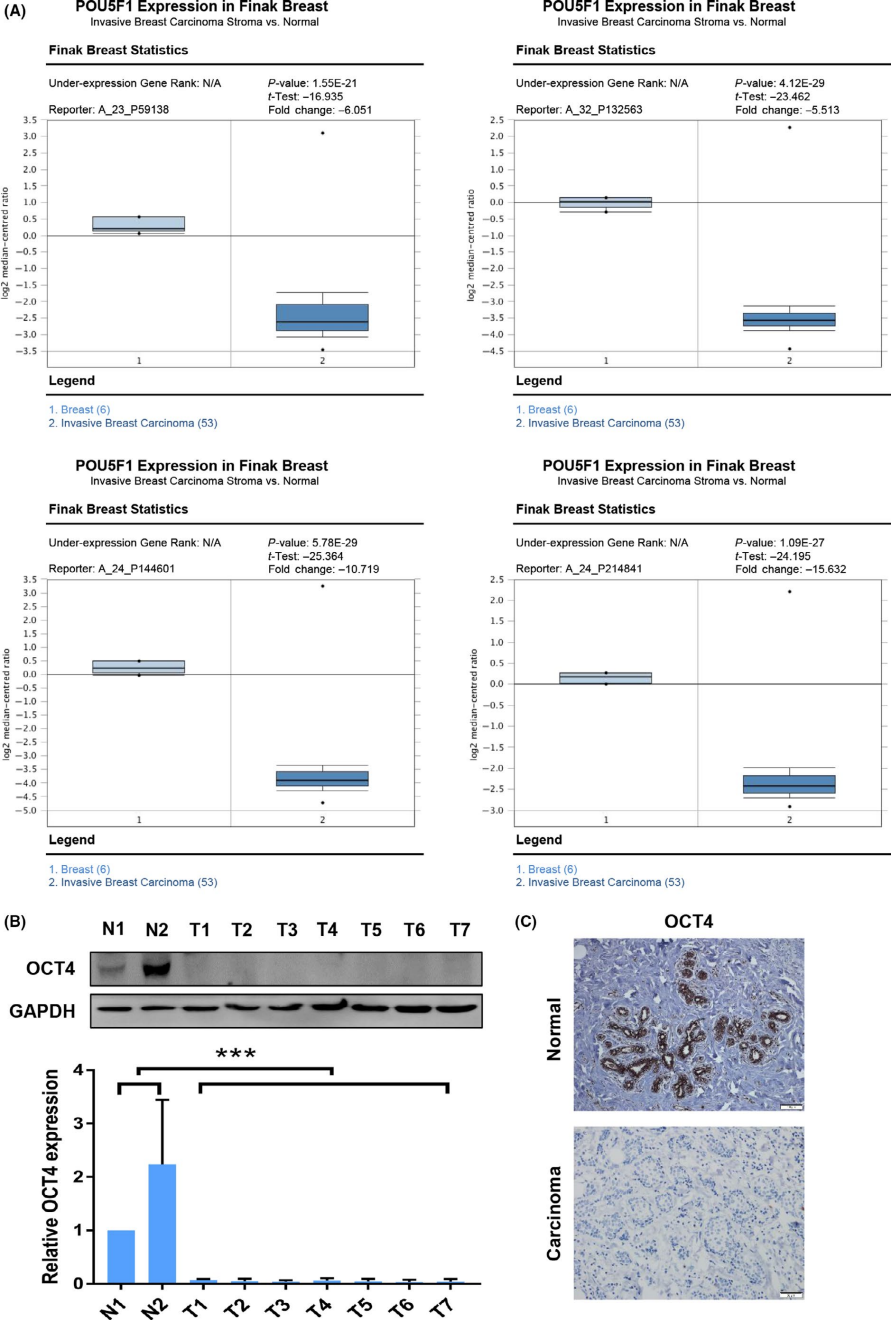
OCT4 expression was down‐regulated in breast cancer compared with normal breast samples. (A) OCT4 mRNA expression was significantly decreased from breast to invasive breast carcinoma in all the four probes (A_23_P59138, A_32_P132563, A_24_P144601, A_24_P214841) in the Finak Breast data set as revealed by Oncomine data‐mining analysis. (B) The protein expression of OCT4 in breast normal tissues (N) and breast cancer tissues (T) was further confirmed by Western blot analysis. (C) The expression of OCT4 in breast normal samples and breast cancer samples was detected by Immunohistochemistry (IHC) analysis. Images were taken under a fluorescence microscope at 200× magnification. ***P < 0.001

Previous analyses of OCT4 expression in human somatic tumour cell lines showed that OCT4 expression was absent in MCF‐7 and HeLa cells compared with nTera cells.^25^ In the Finak Breast data set, we found that the *POU5F1* mRNA level was significantly decreased in invasive breast cancer compared to the breast samples with all four probes (Figure 1A). Consistently, OCT4 expression declined in 40 human breast cancer tissues relative to 10 normal breast tissues, as shown by using Western blot and IHC analyses (Figure 1B,C). These results indicate that OCT4 might play an important role in suppressing breast cancer progression.

### 3.2 OCT4 plays an important role in breast cancer proliferation in vitro and in vivo

**Figure 2:**
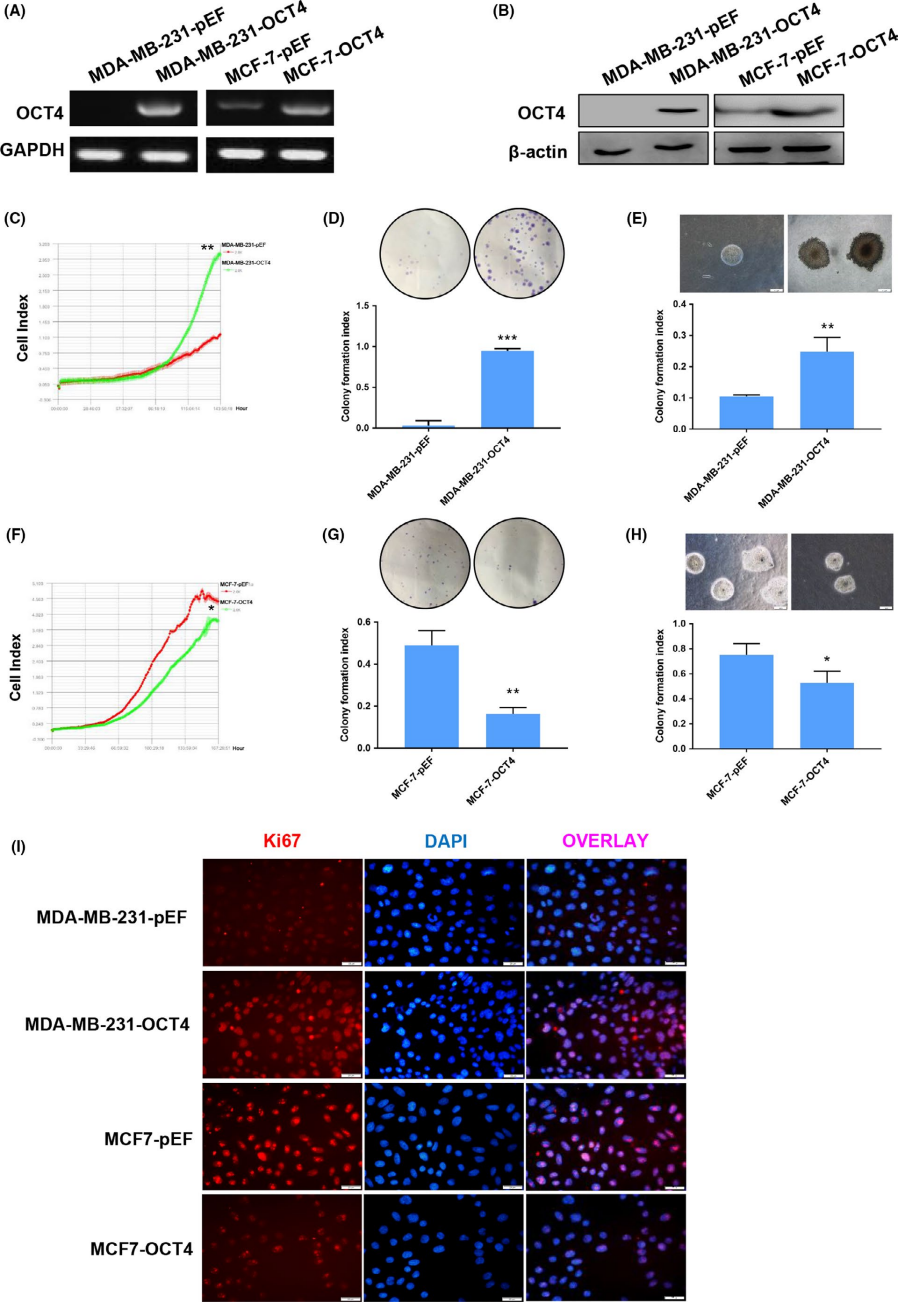
OCT4 plays dual roles in the proliferation of different breast cancer subtypes. (A, B) Using lentivirus transduction, OCT4 was overexpressed in MDA‐MB‐231 and MCF‐7 cells. MDA‐MB‐231 and MCF‐7 cells transduced with empty vector control (MDA‐MB‐231‐pEF, MCF‐7‐pEF) and OCT4 overexpression vector (MDA‐MB‐231‐OCT4, MCF‐7‐OCT4) were analysed by RT‐PCR and Western blot assays. (C‐E) MDA‐MB‐231 cells overexpressing OCT4 were examined for cell proliferation compared to the control group by iCELLigence Real‐Time Cell Analysis system, plate colony formation assay and soft agar colony formation assay. (F‐H) The proliferation of MCF‐7 cells overexpressing OCT4 was compared to the control group using iCELLigence Real‐Time Cell Analysis system, plate colony formation assay and soft agar colony formation assay. (I) Immunofluorescence images of MDA‐MB‐231 and MCF‐7 cells overexpressing OCT4 that showed Ki67 staining (red). DAPI (blue) indicates the cell nucleus. Images were taken under a fluorescence microscope at 200× magnification. Each experiment was repeated three times. *P < 0.05. **P < 0.01. ***P < 0.001

To assess the role of OCT4 in BCCs, we established stable OCT4‐overexpressing MDA‐MB‐231 (TNBC subtype BCC), MCF‐7 (luminal A subtype BCC) and SKBR3 (HER2 subtype BCC) cell lines. We verified the expression of OCT4 by RT‐PCR and Western blot analyses (Figure 2A,B and Figure S1A,B). To eliminate confusion about other isoforms and pseudogenes, we detected *OCT4A*, *OCT4B* and seven *OCT4* pseudogenes expression in breast cancer cells using RT‐PCR. All of the above genes were not expressed in MDA‐MB‐231 cells. *OCT4A*, *OCT4B*, *OCT4‐pg1*, *OCT4‐pg4*, *OCT4‐pg5* and *OCT4‐pg6* were expressed at low levels in MCF‐7 cells, and *OCT4‐pg2*, *OCT4‐pg3* and *OCT4‐pg7* were not expressed in MCF‐7 cells (Figure S2A). Additionally, overexpression of OCT4 increased *OCT4A* and *OCT4‐pg4* expression compared to the control group in MDA‐MB‐231 and MCF‐7 cells (Figure S2B).

**Figure 3:**
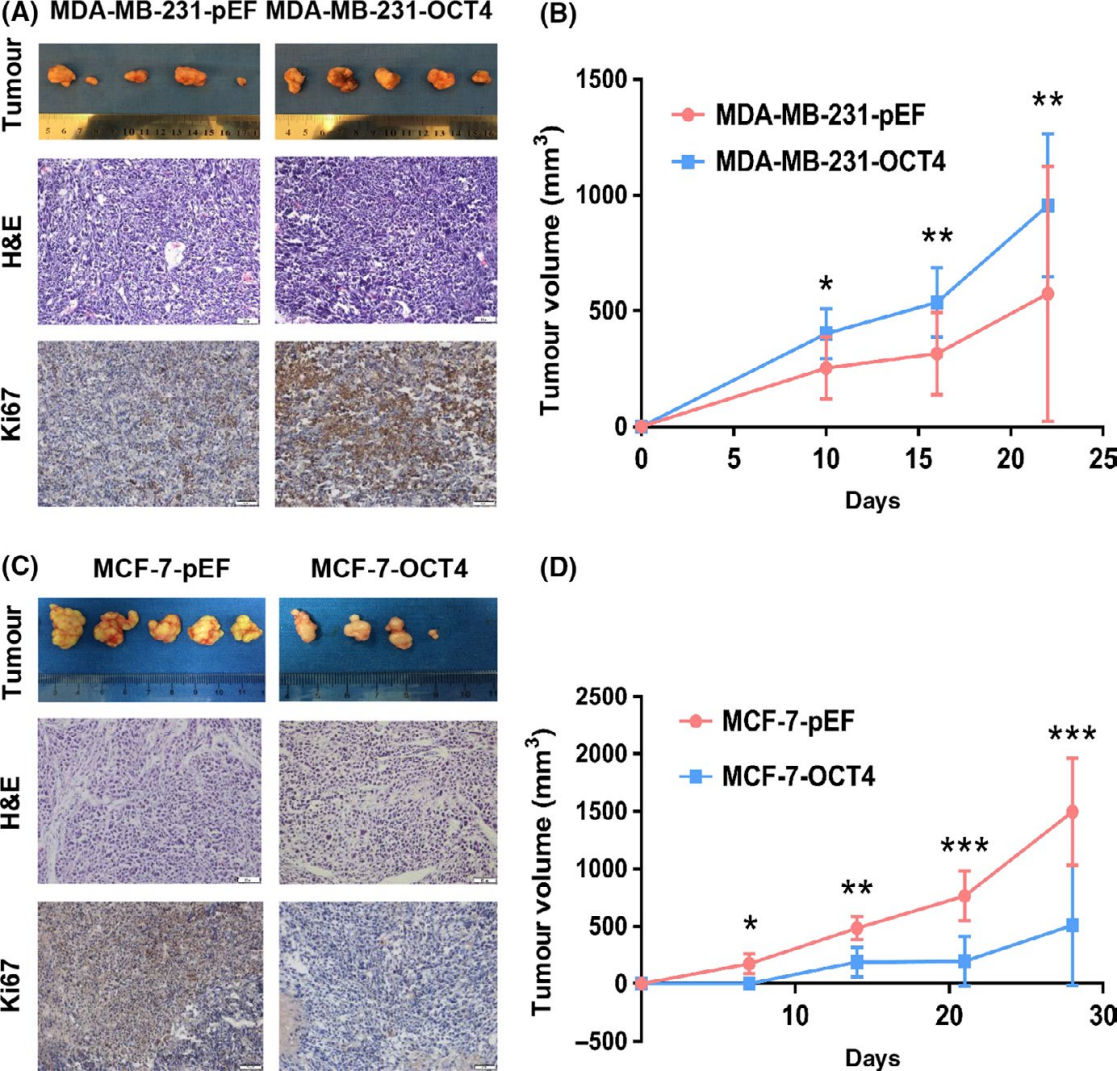
OCT4 regulates the proliferation of breast cancer cells (BCCs) in vivo. (A) Subcutaneous transplant tumour models of BALB/c nude mice were established using MDA‐MB‐231 cells transduced by OCT4 overexpression and empty vectors. Images showed the tumours that were dissected from the mice. Each group had five mice. H&E and Ki67 staining images were sections of tumours. Images were taken under a light microscope at 400× magnification. (B) Tumour growth was quantified. (C) MCF‐7 cells overexpressing OCT4 and the control group were subcutaneously injected into BALB/c nude mice. Images showed the dissected tumours from the mice. Each group showed 5 mice. H&E and Ki67 staining images were sections of tumours. Images were taken under a light microscope at 200× magnification. (D) Tumour growth was quantified. *P < 0.05. **P < 0.01. ***P < 0.001

Then, the effects of OCT4 on cell proliferation were first detected. iCELLigence Real‐Time Cell Analysis system, plate colony formation assay and soft agar colony formation assay showed that OCT4 overexpression dramatically promoted the proliferation of MDA‐MB‐231 (Figure 2C‐E) and SKBR3 cells (Figure S3A,B). However, OCT4 overexpression suppressed MCF‐7 cells' proliferation (Figure 2F‐H). We next determined Ki67 expression (proliferation marker) in MDA‐MB‐231 and MCF‐7 cells by immunofluorescence assay (Figure 2I). The results showed that Ki67 expression was higher in MDA‐MB‐231‐OCT4 cells than the control group and lower in MCF‐7‐OCT4 cells than the control group. Then, we addressed the effect of OCT4 on MDA‐MB‐231 and MCF‐7 cells in vivo. Nude mice were subcutaneously injected with MDA‐MB‐231‐pEF (control group), MDA‐MB‐231‐OCT4, MCF‐7‐pEF (control group) and MCF‐7‐OCT4 cells, and they were monitored for tumour development. Interestingly, the tumour volume was increased in the OCT4‐induced MDA‐MB‐231 group compared with the control group, and the vast majority of subcutaneous tumours were strongly positive for Ki67 in the OCT4‐induced MDA‐MB‐231 group, indicating that OCT4 facilitated tumour formation of MDA‐MB‐231 cells (Figure 3A,B). However, the tumour volume of the OCT4‐induced MCF‐7 group was decreased compared with the control group, and the positive expression for Ki67 was decreased in OCT4‐induced MCF‐7 cells (Figure 3C,D). Collectively, these findings demonstrate that OCT4 plays opposite roles in the tumour‐propagating capacity of TNBC and luminal subtype cells in vitro and in vivo.

### 3.3 OCT4 effects on DNMT1 and Ras/Raf1/ERK1/2 signalling pathway

**Figure 4:**
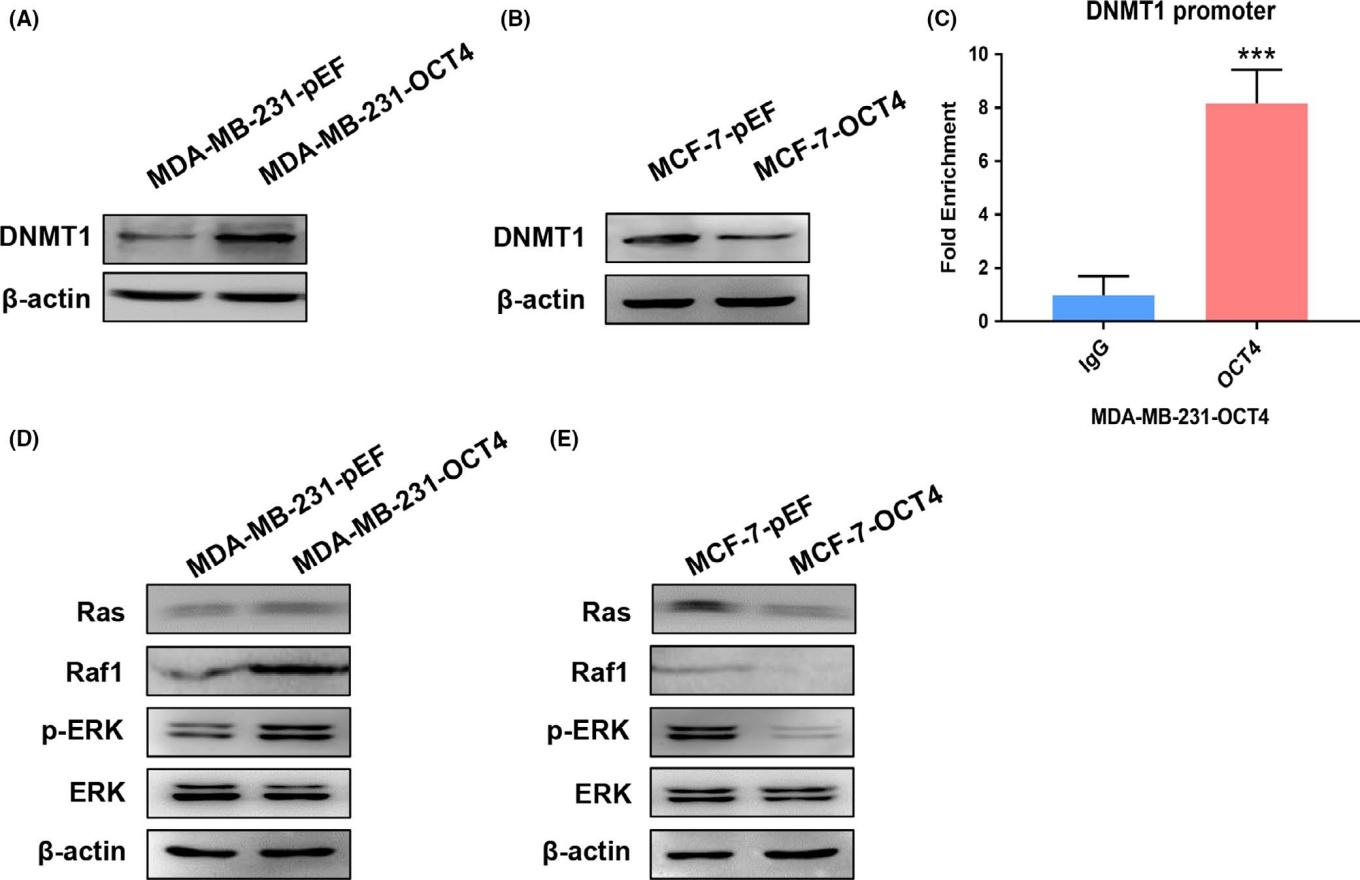
OCT4 regulates DNMT1 expression and Ras/Raf1/ERK signalling pathway in BCCs. (A, B) Using Western blot analysis, DNMT1 expression was analysed in MDA‐MB‐231 and MCF‐7 cells transduced by OCT4 overexpression and empty vectors, respectively. (C) Chromatin immunoprecipitation (ChIP) assays were performed to determine OCT4 binding to the promoter of DNMT1 in MDA‐MB‐231‐OCT4 cells. (D, E) The expression levels of Ras, Raf1, p‐ERK and ERK were analysed by Western blot in MDA‐MB‐231 and MCF‐7 cells transduced by OCT4 overexpression and empty vector. ***P < 0.001

As OCT4 is involved in epigenetic regulation in embryonic stem cells, we analysed DNA methylation. DNA methylation, mediated by the combined action of three DNMTs (DNMT1, DNMT3a and DNMT3b), is associated with tumour initiation and progression.^12^ Western blot analysis showed that overexpression of OCT4 up‐regulated DNMT1 expression in MDA‐MB‐231 cells (Figure 4A), whereas OCT4 overexpression in MCF‐7 cells dramatically down‐regulated DNMT1 expression (Figure 4B). Quantitative chromatin immunoprecipitation (ChIP) showed that OCT4 strongly binds to the proximal region of the DNMT1 promoter in MDA‐MB‐231‐OCT4 cells (Figure 4C). Accordingly, we detected extracellular signal‐regulated kinase (ERK) signalling pathway proteins (markers of cell proliferation).^26^, ^27^ OCT4 activated the ERK signalling pathway in MDA‐MB‐231 and SKBR3 cells (Figure 4D and Figure S4), whereas OCT4 inactivated the pathway in MCF‐7 cells (Figure 4E). These findings indicate that OCT4 may affect the proliferation of BCCs through DNMT1 and ERK signalling pathway.

### 3.4 5‐aza‐dC and zebularine inhibit MDA‐MB‐231‐OCT4 cell proliferation via inactivation of ERK signalling pathway

**Figure 5:**
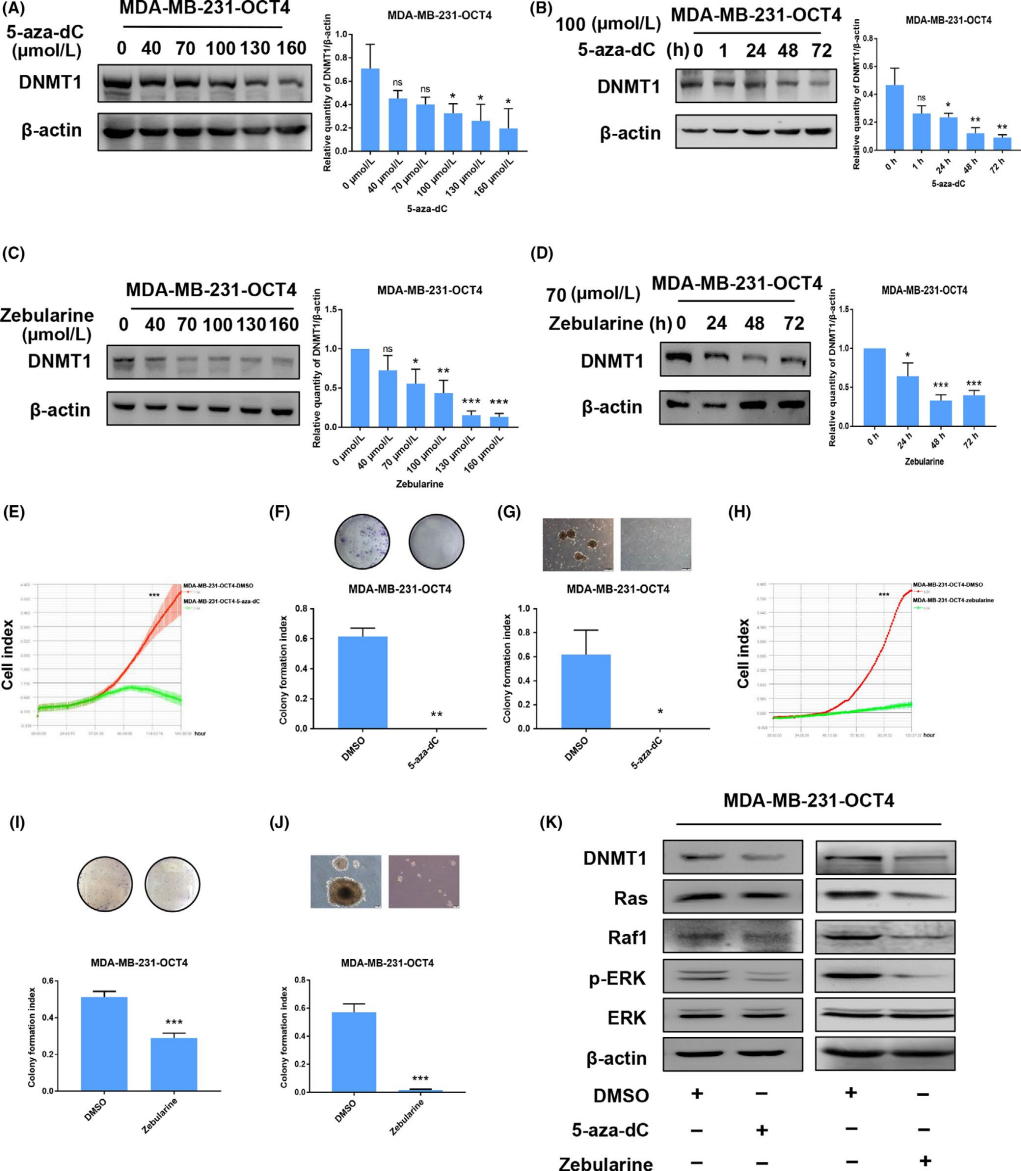
5‐aza‐dC and zebularine suppress the proliferative ability of MDA‐MB‐231‐OCT4 cells via the inactivation of ERK signalling pathway. (A, B) Western blot analysis was used to show that 5‐aza‐dC application inhibited DNMT1 expression in a concentration‐ and time‐dependent manner in MDA‐MB‐231‐OCT4 cells. (C, D) Zebularine application inhibited DNMT1 expression in a concentration‐ and time‐dependent manner in MDA‐MB‐231‐OCT4 cells as shown by Western blot. (E‐G) Treating MDA‐MB‐231‐OCT4 with 5‐aza‐dC (100 μmol/L) suppressed proliferation in iCELLigence Real‐Time Cell Analysis system, plate colony formation assay and soft agar colony formation assay. (H‐J) Treating MDA‐MB‐231‐OCT4 with zebularine (70 μmol/L) suppressed proliferation in iCELLigence Real‐Time Cell Analysis system, plate colony formation assay and soft agar colony formation assay. (K) The expression levels of DNMT1, Ras, Raf1, p‐ERK and ERK were detected in MDA‐MB‐231‐OCT4 cells treated with 5‐aza‐dC (100 μmol/L for 72 h) and zebularine (70 μmol/L for 48 h) by Western blot. *P < 0.05. **P < 0.01. ***P < 0.001

To investigate whether OCT4 affects cell proliferation by regulating DNMT1 and ERK signalling pathway, we treated MDA‐MB‐231‐OCT4 cells with 5‐aza‐dC and zebularine, which are well characterized and clinically relevant DNMT inhibitors.^28^, ^29^ The IC_50_ for 5‐aza‐dC for 145 hour was 133 μmol/L for MDA‐MB‐231‐OCT4 cells (Figure S5A), and the IC_50_ for zebularine for 96 hour was 92.5 μmol/L for MDA‐MB‐231‐OCT4 cells (Figure S5B). MDA‐MB‐231‐OCT4 cells were treated with varying doses of 5‐aza‐dC and zebularine for various lengths of time and analysed by Western blot assay. Both 5‐aza‐dC and zebularine inhibited DNMT1 in a dose‐ and time‐dependent manner in MDA‐MB‐231‐OCT4 cells (Figure 5A‐D). Then, we used iCELLigence Real‐Time Cell Analysis system, plate colony formation assay and soft agar colony formation assay to examine the cell proliferation of MDA‐MB‐231‐OCT4 cells treated with 100 μmol/L 5‐aza‐dC for 72 hour and 70 μmol/L zebularine for 48 hour. Both 5‐aza‐dC and zebularine abrogated OCT4 function, promoting the proliferation of MDA‐MB‐231 cells (Figure 5E‐J). Both 5‐aza‐dC and zebularine could inactivate Ras/Raf1/ERK signalling pathway (Figure 5K), demonstrating that OCT4 activates ERK signalling pathway by DNMT1 to facilitate proliferation of MDA‐MB‐231 cells.

### 3.5 ISL1 expression is associated with overall survival in breast cancer

**Figure 6:**
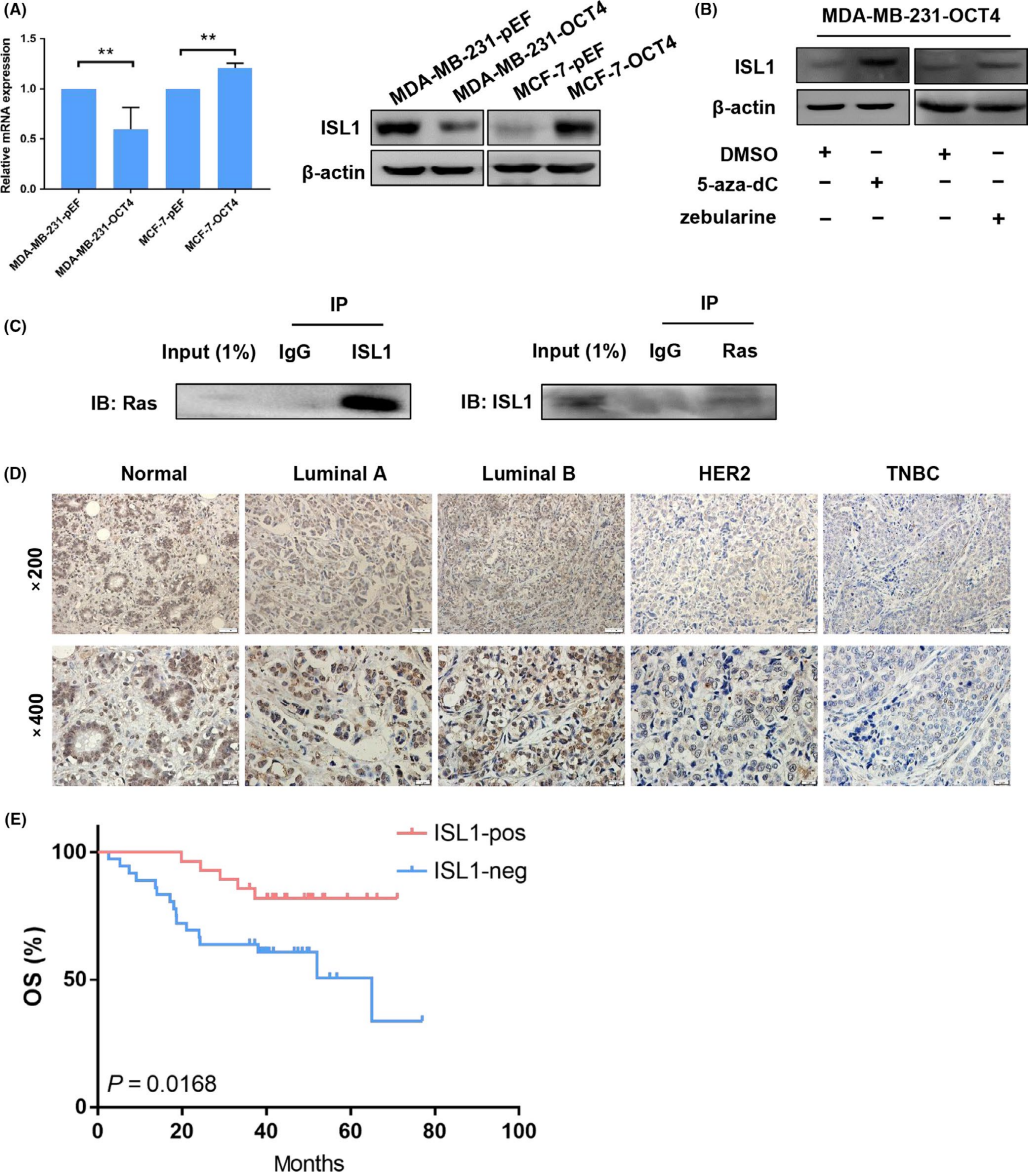
ISL1 interacts with Ras and associates with overall survival in breast cancer. (A) Using PCR and Western blot, ISL1 expression was determined in MDA‐MB‐231 and MCF‐7 cells transduced by OCT4 and empty vector. (B) ISL1 expression was detected in MDA‐MB‐231 OCT4‐induced cells treated with 5‐aza‐dC and zebularine. (C) The interaction of ISL1 and Ras was determined by co‐immunoprecipitation (CoIP) in MCF‐7‐OCT4 cells. (D) IHC staining for ISL1 expression was performed in normal human breast tissues and different human breast cancer subtype tissues. Images were taken under a light microscope at 200× and 400× magnification. (E) The Kaplan‐Meier curves for OS showed positive and negative ISL1 expression. The log‐rank test *P*‐value is shown

We analysed DNMT1 downstream target ISL1, which is considered as a tumour suppressor gene and is hypermethylated in cancer.^30^ As ISL1 is a downstream target of DNMT1 and correlated with BCC proliferation, we analysed ISL1 expression in OCT4‐induced BCCs. Western blot analysis showed that overexpression of OCT4 down‐regulated ISL1 expression in MDA‐MB‐231 cells, whereas OCT4 overexpression up‐regulated ISL1 expression in MCF‐7 cells (Figure 6A). ISL1 expression was restored in MDA‐MB‐231‐OCT4 cells treated with 5‐aza‐dC and zebularine (Figure 6B). Additionally, we found that ISL1 interacted with Ras in MCF‐7‐OCT4 cells (Figure 6C). These findings reveal that DNMT1 inactivates ERK signalling pathway by targeting ISL1 in MDA‐MB‐231 and MCF‐7 cells.

Next, we evaluated ISL1 expression in 10 samples of normal breast tissues and 66 samples of different subtypes of breast cancer tissues. IHC analysis revealed that ISL1 was localized predominantly in the nucleus (Figure 6D). We further investigated the relationship between ISL1 expression and clinical parameters. We found that ISL1 expression was correlated to tumour size, molecular subtype, ER, PR, HER2 and Ki67 status (Table 2). Moreover, we constructed Kaplan‐Meier curves for the overall survival (OS), which indicated that patients with ISL1‐positive tumours had a higher OS rate (Figure 6E). These results demonstrate that ISL1 is a tumour suppressor gene in BC and may be associated with tumorigenesis and progression in BC.

**Table 2:** Association of ISL1 expression with clinicopathological parameters of breast cancer patients

Characteristics	No.	ISL1 expression (%)		*P* value
		Positive	Negative	
Age (y)				
<50	18	9 (50)	9 (50)	0.446
≥50	48	19 (39.6)	29 (60.4)	
Size (cm)				
≤2	34	21 (61.76)	13 (38.24)	0.002**
2‐5	29	5 (17.24)	24 (82.76)	
>5	3	1 (33.33)	2 (66.67)	
Lymph node status				
Negative	36	18 (50)	18 (50)	0.538
1‐3	11	4 (36.36)	7 (63.64)	
4‐9	11	4 (36.36)	7 (63.64)	
>9	8	2 (25)	6 (75)	
Molecular subtype				
Luminal A	15	13 (86.67)	2 (13.33)	0.000***
Luminal B	15	11 (73.33)	4 (26.67)	
HER2‐enriched	18	1 (5.56)	17 (94.44)	
Triple‐negative	18	3 (16.67)	15 (83.33)	
ER status				
Negative	36	4 (11.11)	32 (88.89)	0.000***
Positive	30	24 (80)	6 (20)	
PR status				
Negative	37	4 (10.81)	33 (89.19)	0.000***
Positive	29	24 (82.76)	5 (17.24)	
HER2 status				
Negative	41	22 (53.66)	19 (46.34)	0.018*
Positive	25	6 (24)	19 (76)	
Ki67 status				
≤20%	28	18 (64.29)	10 (35.71)	0.002**
>20%	38	10 (26.32)	28 (73.68)	

Significance of association was determined using Pearson's chi‐square (*χ* ^2^) test.

^**^ *P* = 0.01, ^***^ *P* = 0.001.

### 3.6 OCT4 interacting with ERα suppresses cell proliferation of MCF‐7 cells

**Figure 7:**
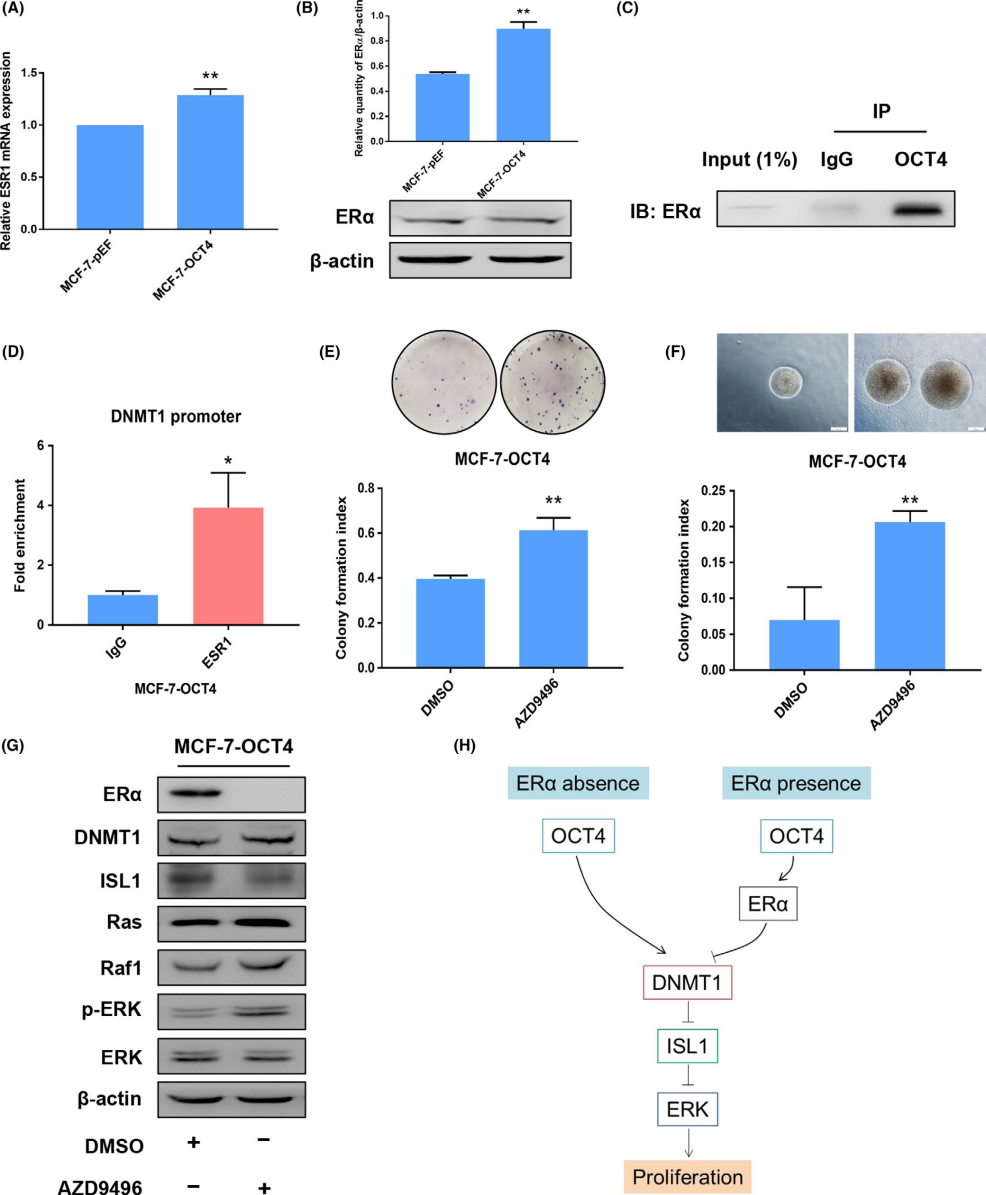
OCT4 and ERα complexes inhibit cell proliferation of MCF‐7 cells via DNMT1/ISL1/ERK axis. (A) ESR1 expression was detected by RT‐PCR in MCF‐7 cells transduced by OCT4 and empty vector. (B) ERα expression was detected by Western blot in MCF‐7 cells transduced by OCT4 and empty vector. (C) In CoIP assays, OCT4 interacted with ERα in MCF‐7‐OCT4 cells. (D) ChIP assay was performed to analyse ERα binding to the ERE‐containing promoter of DNMT1 in MCF‐7‐OCT4 cells. (E, F) Treating MCF‐7‐OCT4 with AZD9496 (300 nmol/L) suppressed proliferation, as shown by plate colony formation assay and soft agar colony formation assay. (G) The expression levels of ERα, DNMT1, ISL1, Ras, Raf1, p‐ERK and ERK were detected by Western blot in MCF‐7‐OCT4 cells that were treated with AZD9496 (300 nmol/L for 1 h). (H) In MDA‐MB‐231 cells (ERα absence), OCT4 binding to the DNMT1 promoter promoted cell proliferation through the down‐regulation of ISL1 expression and the activation of ERK signalling pathway. The interaction of OCT4 and ERα affected the transcriptional activity of DNMT1 to suppress MCF‐7 cell (ERα presence) proliferation through up‐regulation of ISL1 expression and inactivation of ERK signalling pathway. *P < 0.05. **P < 0.01

To elucidate the mechanism of the dual functions of OCT4 in proliferation of different subtypes of BCC, we hypothesized that the opposing roles of OCT4 in cell proliferation might be associated with the intrinsic characteristics of BCCs. Previous studies showed that DNMT1 was negatively correlated with ERα in breast cancer.^31^ Therefore, we tested ERα expression in MDA‐MB‐231‐OCT4 and MCF‐7‐OCT4 cells by using PCR and Western blot analyses. ERα expression was undetectable in both MDA‐MB‐231‐pEF and MDA‐MB‐231‐OCT4 cells (Figure S6), whereas ERα expression was significantly up‐regulated in MCF‐7‐OCT4 cells (Figure 7A,B). Co‐Immunoprecipitation (CoIP) assay revealed that OCT4 interacted with ERα in MCF‐7‐OCT4 cells (Figure 7C). ChIP showed that ERα strongly binds to the proximal region of the DNMT1 promoter in MCF‐7‐OCT4 cells (Figure 7D). Subsequently, we treated MCF‐7‐OCT4 cells with the ERα antagonist AZD9496. According to recent research, AZD9496 inhibits MCF‐7 cell growth.^32^, ^33^, ^34^ However, AZD9496 dramatically promoted the proliferation of MCF‐7‐OCT4 cells using plate colony formation assay and soft agar colony formation assay (Figure 7E,F). Consistent with our hypothesis, AZD9496 induced DNMT1 expression, down‐regulated ISL1 expression and activated ERK signalling pathway in MCF‐7‐OCT4 cells (Figure 7G). These results demonstrate that OCT4 overexpression with the loss of ERα promoted the proliferation of breast cancer cells, indicating that OCT4 is dependent on ERα to suppress the proliferation of breast cancer cells through DNMT1/ISL1/ERK axis (Figure 7H).

## 4 DISCUSSION

A review of the published literature reveals that OCT4 is absent in somatic tumour cell lines, indicating its tumour‐suppressive effects. However, the link between OCT4 and tumour progression has been poorly investigated. In this study, our analysis of human breast cancer data reveals that OCT4 is down‐regulated in breast cancer relative to the normal breast tissue. OCT4 induced ERα expression in MCF‐7 cells and interacted with ERα to downregulate DNMT1 expression by ERα binding to the promoter region. We found that OCT4 regulated DNMT1 expression, accompanied by ISL1 expression and activation of ERK proliferative signalling pathway. We further provided the mechanistic insight into the role of OCT4 in suppression of MCF‐7 cell proliferation. Inhibition of ERα by AZD9496 in MCF‐7‐OCT4 cells facilitated MCF‐7 cell proliferation and re‐activated Ras/Raf1/ERK signalling pathway. Thus, for the first time, we demonstrate that OCT4 is dependent on ERα expression to suppress proliferation in ERα‐positive breast cancer cells through the regulation of DNMT1/ISL1 and the inactivation of ERK signalling pathway.

The transcription factor OCT4 is a stem cell marker that functions in the maintenance of self‐renewal and pluripotency. OCT4 regulates target genes to play the opposite roles of OCT4 in tumorigenesis and cancer progression, which is a complicated process. Recent studies show that OCT4 may correlate with cancer progression. However, the majority of adult somatic tumour cells are differentiated without the capacity for self‐renewal. OCT4 expression is lost upon additional differentiation and maturation,^35^ associated with DNA methylation of OCT4.^36^ Additionally, in human teratoma cells (OCT4 positive control), the distal enhancer region of OCT4 was found to be completely unmethylated (0.0%), whereas in HeLa and MCF‐7 cells, the promoter region was highly methylated (92.3% and 98.7%, respectively), suggesting that expression of OCT4 was completely silenced in both of these cell lines.^25^ Loss of OCT4 in breast cancer may predominantly correlate with epigenetic mechanisms, such as DNA methylation. OCT4 has oncogenic and tumour suppressor activities in BCCs.^5^, ^7^, ^37^ OCT4 expression in human somatic tumours remains controversial. Consistent with tumour‐suppressive function, OCT4 has been shown to suppress the metastatic potential of BCCs via Rnd1 down‐regulation, while OCT4 overexpression leads to the up‐regulation of E‐cadherin expression, even in BCCs with high E‐cadherin levels.^5^ Conversely, OCT4 is also believed to facilitate cancer progression by increasing BIRC5 and CCND1 expression in hepatocellular carcinoma.^38^ OCT4 promotes drug resistance and metastasis in lung cancer by regulating downstream PTEN and TNC genes.^8^ The human OCT4 gene generates three variants. *OCT4A* is not expressed in breast cancer cells, such as MDA‐MB‐231 and MCF‐7 cells.^39^ *OCT4B* is amplified and promotes an aggressive phenotype in gastric cancer.^40^ Moreover, the OCT4 pseudogenes *OCT4‐pg1* and *OCT4‐pg5* were found in somatic cancers.^2^ *OCT4‐pg4* is positively correlated with *OCT4* in hepatocellular carcinoma, OCT4‐induction may promote *OCT4‐pg4* expression in BCCs.^41^ Remarkably, OCT4 pseudogenes did not show OCT4A activities. Thus, this controversy may be related to the interferences of OCT4B and OCT4 pseudogenes.

Epigenetic silencing of tumour suppressors by CpG island hypermethylation is a common hallmark of cancer,^42^ and OCT4 directly binds to the promoter of DNMT1 and enhances its expression in mesenchymal stem cells.^43^ DNMT1 is required to maintain DNA methylation patterns in mammalian cells and is thought to be the predominant maintenance methyltransferase gene.^44^ The estrogen receptor alpha (ERα) is considered to be involved in breast cancer progression.^45^ ERα modulates transcription by forming complexes with other proteins and then binding to the estrogen response elements (EREs). The POU domain interacts with the DNA‐binding domain of the ER, and this interaction also affects the transcriptional activity of an ERE‐containing promoter.^46^ The DNMT1 promoter includes ERE, indicating that ESR1 may regulate DNMT1 by binding to the promoter. Conversely, specific inhibition of DNMT1 induces re‐expression of ERα in ERα‐negative human breast cancer cells.^47^ Both the DNMT1 inhibitors 5‐aza‐dC and zebularine reactivate functional ERα expression in MDA‐MB‐231 cells.^29^, ^48^ Furthermore, ERα can specifically interact with ISL1 in the female rat hypothalamus.^49^ However, the relationship between ERα and ISL1 requires further investigation.

In summary, this study demonstrates that OCT4 is involved in tumour‐proliferative capacity by modulating DNMT1/ISL1 expression and ERK signalling pathway. We propose that OCT4 suppresses BCC proliferation in an ERα‐dependent manner. Our current work shows that OCT4 requires ERα to suppress the proliferation of breast cancer cells through the DNMT1/ISL1/ERK axis, providing a novel molecular circuit for inhibiting BCC proliferation. Moreover, we proposed that OCT4 functions as both a tumour suppressor and a tumour promoter, mediating tumour suppressor functions in early tumorigenesis and ERα‐positive breast cancer progression, and tumour promoter functions in ERα‐negative breast cancer progression. OCT4 expression level may provide potential clinical diagnostic biomarker in breast cancer. Our data could also contribute to developing new molecular target therapies for patients with ERα‐positive breast cancer. However, other molecular players that participate in this process may exist, and similar underlying patterns require further investigation.

## CONFLICT OF INTEREST

The authors declare no conflict of interest.

## AUTHOR CONTRIBUTION

C.Q., X.J. and Y.Li. were involved in the study conception and design. Y.G., Y.J., X.Z. and H.Q. participated in data acquisition and analysis. Y.Lu., P.S., Y.S., D.Q. and W.X. performed the experiments. X.J. drafted and approved the article. C.Q. revised the article critically. All authors have read and approved the manuscript for publication.
