# Supplementary material for: ERα is required for suppressing OCT4‐induced proliferation of breast cancer cells via DNMT1/ISL1/ERK axis
Source: Cell Prolif. 2019 Apr 22;52(4):e12612. doi: 10.1111/cpr.12612 (PMC6668970; doi:10.1111/cpr.12612)
Supplement: Supplementary file 1 [file CPR-52-e12612-s001.docx]

**Supplementary files**

Figure S1


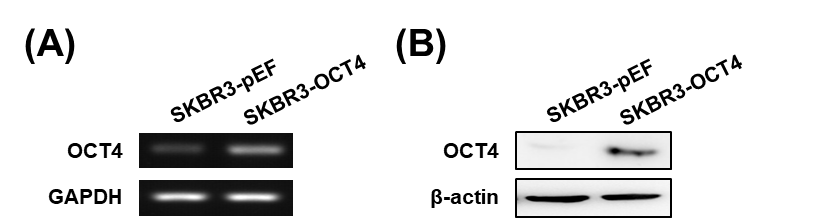


**FIGURE S1.** (A, B) Using lentivirus transduction, OCT4 was overexpressed in SKBR3 cells. SKBR3 cells transduced with empty vector control (SKBR3-pEF) and overexpression of OCT4 (SKBR3-OCT4) were detected by RT-PCR and Western blot.

Figure S2


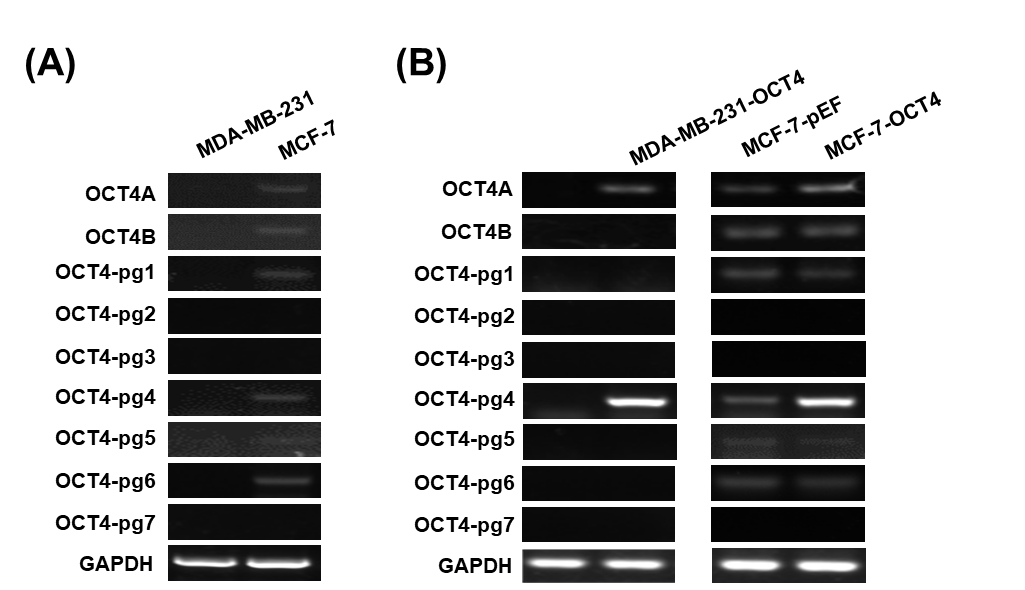


**FIGURE S2.** (A) *OCT4A*, *OCT4B*, *OCT4-pg1*, *OCT4-pg2*, *OCT4-pg3*, *OCT4-pg4*, *OCT4-pg5*, *OCT4-pg6* and *OCT4-pg7* expression was detected in MDA-MB-231 and MCF-7 cells using RT-PCR assays. (B) *OCT4A*, *OCT4B*, *OCT4-pg1*, *OCT4-pg2*, *OCT4-pg3*, *OCT4-pg4*, *OCT4-pg5*, *OCT4-pg6* and *OCT4-pg7* expression was detected by RT-PCR assay in MDA-MB-231-OCT4 and MCF-7-OCT4 cells compared to the control group.

Figure S3


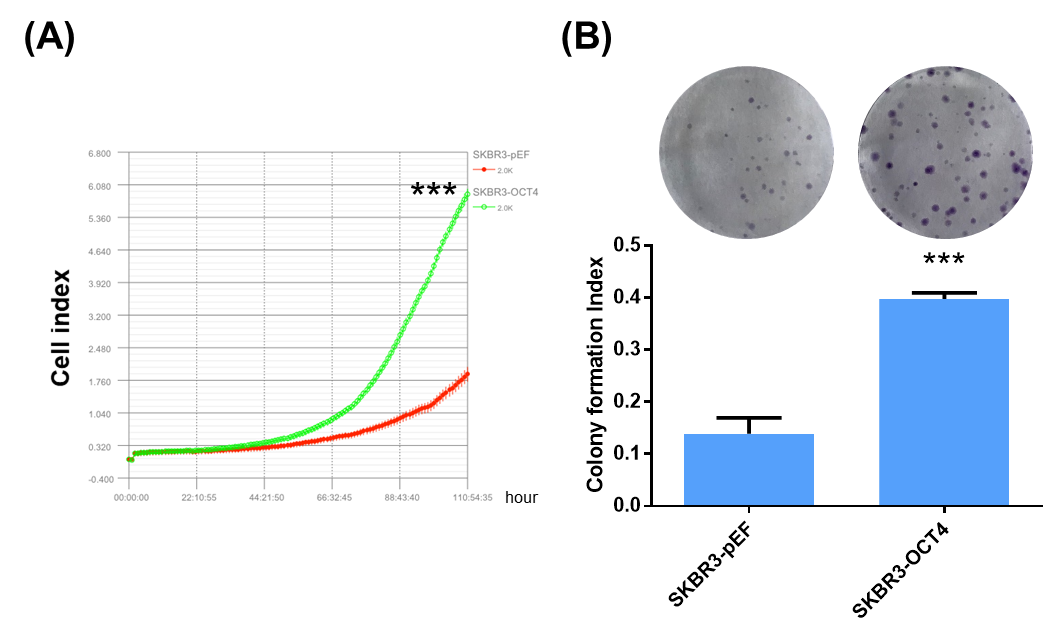


**FIGURE S3.** (A, B) SKBR3 cells overexpressing OCT4 were examined for cell proliferation compared to the control group by iCELLigence Real-Time Cell Analysis system and plate colony formation assay.

Figure S4


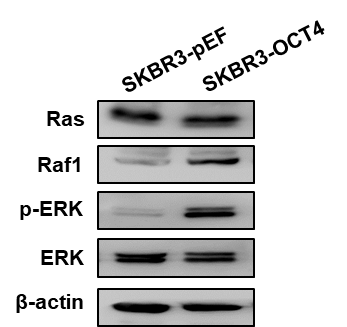


**FIGURE S4.** The expression levels of Ras, Raf1, p-ERK and ERK were analysed in SKBR3 cells transduced by OCT4 overexpression and empty vectors by Western blot analysis.

Figure S5


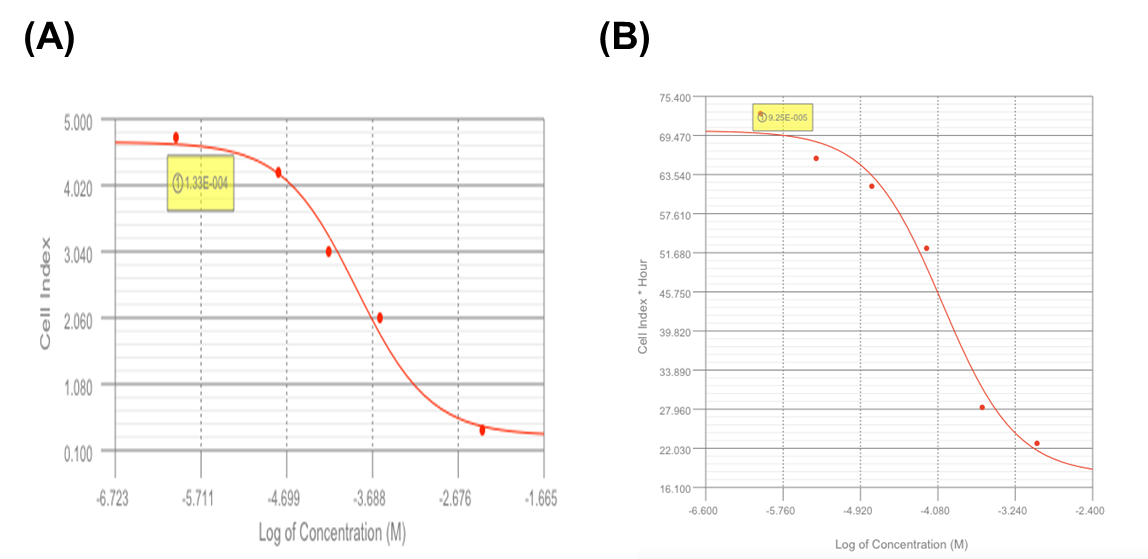


**FIGURE S5.** (A) The IC50 for 5-aza-dC was detected in MDA-MB-231-OCT4 cells by the iCELLigence Real-Time Cell Analysis system. (B) The IC50 for zebularine was detected in MDA-MB-231-OCT4 cells by the iCELLigence Real-Time Cell Analysis system.

Figure S6


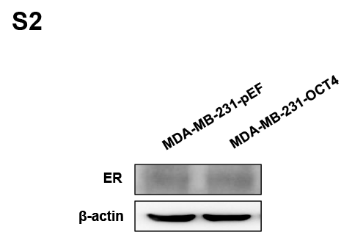


**FIGURE S6.** The expression of ERα was analysed in MDA-MB-231 cells transduced by OCT4 overexpression and empty vectors by western blot.

**Table S1** PCR primers sequences.

| Gene name | Sequence 5’→3’ |
| --- | --- |
| **RT-PCR primers** |  |
| *OCT4A* |  |
| Sense | TGGGCCAGGCTCTGAGGTGT |
| Antisense | TCCTGCTTCGCCCTCAGGCT |
| *OCT4B* |  |
| Sense | CAGGGAATGGGTGAATGAC |
| Antisense | AGGCAGAAGACTTGTAAGAAC |
| *OCT4-pg1* |  |
| Sense | CATGCATGCCCGAAAGAGAAAGCTAT |
| Antisense | TGTGGCTGATCTGCAGTGTGGG |
| *OCT4-pg2* |  |
| Sense | GTGTACATGTTTATAAAGTTTGTGGTAGTGTTC |
| Antisense | GGGTCGCTAGGTAATTTTGTCACTGG |
| *OCT4-pg3* |  |
| Sense | CTTCTCACCCCCTCCAGGC |
| Antisense | CCACTGCTTGATCGCTTGC |
| *OCT4-pg4* |  |
| Sense | GGGACACCTGGCTTCGGATG |
| Antisense | CCCCACACCTCAGAGCCTGA |
| *OCT4-pg5* |  |
| Sense | CAGTGATTATGCACCATGAGAGGA |
| Antisense | GGGAAAGGCACTAAGGAACACAG |
| *OCT4-pg6* |  |
| Sense | CCTAGCAAAACCTCAACGAGTCCCAG |
| Antisense | CAAGAGGTTGTGGTGAGCGAAGG |
| *OCT4-pg7* |  |
| Sense | GCCAAATTGCTGAAGCAGAAGGATATC |
| Antisense | CTGGTCTTGTGAAAGGATACTCAGTGG |
